# Abdominal Aortic Aneurysm: Roles of Inflammatory Cells

**DOI:** 10.3389/fimmu.2020.609161

**Published:** 2021-02-03

**Authors:** Zhen Yuan, Yi Lu, Jia Wei, Jiaqi Wu, Jin Yang, Zhejun Cai

**Affiliations:** ^1^ Department of Cardiology, The Second Affiliated Hospital, Zhejiang University School of Medicine, Hangzhou, China; ^2^ Department of Urology, Children’s Hospital, Zhejiang University School of Medicine, Hangzhou, China; ^3^ Translational Medicine Center, The Affiliated Hospital of Hangzhou Normal University, Hangzhou, China; ^4^ Institute of Hepatology and Metabolic Diseases, Hangzhou Normal University, Hangzhou, China; ^5^ Jiaxing Key Laboratory of Cardiac Rehabilitation, Jiaxing, China

**Keywords:** abdominal aortic aneurysm, inflammation, T cells, macrophages, inflammasome, neutrophil extracellular traps

## Abstract

Abdominal aortic aneurysms (AAAs) are local dilations of infrarenal segment of aortas. Molecular mechanisms underlying the pathogenesis of AAA remain not fully clear. However, inflammation has been considered as a central player in the development of AAA. In the past few decades, studies demonstrated a host of inflammatory cells, including T cells, macrophages, dendritic cells, neutrophils, B cells, and mast cells, etc. infiltrating into aortic walls, which implicated their crucial roles. In addition to direct cell contacts and cytokine or protease secretions, special structures like inflammasomes and neutrophil extracellular traps have been investigated to explore their functions in aneurysm formation. The above-mentioned inflammatory cells and associated structures may initiate and promote AAA expansion. Understanding their impacts and interaction networks formation is meaningful to develop new strategies of screening and pharmacological interventions for AAA. In this review, we aim to discuss the roles and mechanisms of these inflammatory cells in AAA pathogenesis.

## Introduction

Abdominal aortic aneurysm (AAA) is one of the most common types of true aneurysms in the world. AAA is defined when the maximal abdominal aortic diameter reaches 30 mm or 1.5 times of the normal ones. The estimated AAA prevalence in men aged over 60 years is about 4–8%, and the prevalence in women gets 0.5–1.5% or so (1). The major risk factors of AAA include cigarette smoking, aging, male gender and corresponding family history (2, 3). The most common cause of death for AAA patients is aneurysm rupture, which accounts for an approximately 60% of mortality (4).

In the past decades, AAA has been regarded as a result of long-term atherosclerotic lesions, which shares the same pathogenesis with other cardiovascular diseases (CVD), due to similar risk factors such as male sex, tobacco consumption, family history, hyperlipidemia and elder population (5, 6). However, diabetes mellitus (DM), a common comorbidity of atherosclerotic disease, is conversely related to AAA development. Patients with DM have a reduction of morbidity by nearly 30 percent (7). Besides, in contrary to the infrarenal segment of aorta, which is the most commonly involved part of AAA, the external iliac artery is often aneurysm-resistant, but it is strongly vulnerable to atherosclerotic occlusive disease (8). Another phenomenon is that the lipid profiles of patients with AAA are not always abnormal like other CVD patients. These findings indicate that the atherosclerotic lesion may be independent of AAA formation.

Recent studies suggest the pathophysiology of AAA is a multifactorial process consisting of inflammation responses, matrix metalloproteinase (MMP) activation, oxidative stress, intraluminal thrombus, smooth muscle apoptosis and extracellular matrix (ECM) degeneration (9–11). The proteases secreted by inflammatory cells can induce degradation of ECM. In the meanwhile, due to destruction of ECM structure and loss of resistance of tunica media, soluble blood components like inflammatory cells are transferred and accumulated in tunica media through the highly vascularized adventitia, resulting in infiltration of inflammatory cells into the vascular media. These processes together with platelet accumulation and coagulation system activation promote intraluminal thrombosis, and subsequently causes aortic dilation and increased vulnerability to AAA rupture (12). Intraluminal thrombosis is capable to create an inflammatory microenvironment containing neutrophils, cytokines, proteases, and reactive oxygen species, and thereby decrease aortic wall strength. These phenomena indicate that inflammatory cells are in the central position of the whole process. This review is an update of recent advances of inflammatory cell-related mechanisms during AAA development.

## Inflammatory Microenvironment

The aortic wall can be generally divided into three layers: tunica adventitia, tunica media and tunica intima, of which tunica adventitia is fully vascularized and permit leukocyte diapedesis. The aortic wall inflammation is characterized as a multicellular-participating process including mononuclear cell infiltration, immunoglobulin (Ig) secretion and cytokine production, suggesting that both innate and adaptive immune responses are involved (13). The histological specimen of human aortic aneurysm tissue reveals that there were a variety of inflammatory cells gathering in the aortic wall. Recent studies showed that perivascular adipose tissue (PVAT) played an essential role in the process of in leukocyte infiltration. When the vascular damage initiates, PVAT increases its volume and then upregulates the expression of inflammatory factors such as resistin, leptin, cytokines and chemokines (14), which induce infiltration of inflammatory cells, including neutrophils, macrophages, natural killer cells (NK cells), dendritic cells (DCs), T and B lymphocytes and mast cells. All these inflammatory cells are implicated in the formation of AAA (13), and the interactions among them formed the inflammatory microenvironment of aortic walls. For example, cytokines secreted by T cells are essential for macrophage activation, while DCs and macrophages can present antigens to T cells to stimulating primary T cell responses (15). Decreasing the activity of inflammatory cells may be a therapeutic strategy to treat non-ruptured AAAs. Daphnetin was recently proved to be eligible to suppress AAA generated with elastase by reducing the infiltration and accumulation of inflammatory cells such as macrophages, T cells and B cells (16). In addition, suppressing the infiltration of CD11b^+^ macrophage and CD4^+^ T cell with antagonism of toll-like receptor 2 significantly ameliorated CaCl_2_-induced aneurysms (17). The fact that animals can benefit from inhibitors of inflammatory cells independent of models proved the central role of these cells in pathogenesis of AAA.

## Innate Immune Cells

### Macrophages

There are generally two origins of macrophages involved in the pathogenesis of AAA: tissue-resident macrophages arising from embryonic precursors, and monocyte-differentiated macrophages from peripheral blood (18). Single-cell RNA sequencing has revealed markedly expansion and activation of aortic resident macrophages, blood-derived monocytes and inflammatory macrophages in the samples of elastase-induced AAA models (19). Tissue-resident macrophages are self-renewed independently of bone marrow activity and can continuously migrate to peripheral tissues. However, the circulating monocytes are the major origin of macrophages gathering in aortic walls (20).

Circulating monocytes originating from the bone marrow play a critical role in encoding antimicrobial and phagocytosis-related proteins (21). When the local environment undergoes inflammatory changes, blood monocytes can be recruited to the tissue and differentiated into macrophages. In response to different inflammatory stimuli, blood monocytes migrate to the tissue and differentiate into distinct macrophages subgroups, including classically activated macrophages (M1 macrophages) and alternatively activated macrophages (M2 macrophages) (22). This process is termed as macrophage polarization. Interestingly, these two subgroups of macrophages serve almost opposite roles in the pathogenesis of AAA.

M1 macrophages are preferentially located in the tunica adventitia of the aortic wall (20). They can be activated by the stimuli like lipopolysaccharide (LPS) and IFN-γ (23). By upregulating massive inflammatory cytokines including TNF-α, IL­6, IL­12, IL­1β, chemokine (C-C motif) ligand 2, and nitric oxide (NO) (24), M1 macrophages aggravate local inflammation and promote the aortic dilation as well as vascular remodeling. On the other hand, M2 macrophage polarization is typically induced by Th2 cytokines like IL-4 and IL-13 (23, 25). By mobilizing together with mast cells and NK cells, M2 macrophages can regulate angiogenesis, cell recruitment, and collagen deposition (26). With the progression of AAA, the aortic walls undergo a switch from M1 macrophage dominance to M2 macrophage dominance, which reflects a compensatory mechanism of the anti-inflammatory and tissue-repair effect of M2 macrophages (20). The counteracting effects of M1 and M2 macrophages in AAA make them eligible for therapeutic applications to control inflammation and destruction of aortic walls. Cheng et al. introduced Notch receptor inhibitors which upregulated M2 macrophages and downregulated M1 macrophages to *Apoe* ^-/-^ mice with AAA, and identified this intervention remarkably ameliorated progression of AAA (27).

### Neutrophils

Neutrophils are a kind of polymorphonuclear leukocytes, which are consistently generated in the bone marrow from myeloid precursors (28) Neutrophils are one of the most abundant immune effector cells of the human immune system, whose main functions include phagocytosis, degranulation, and formation of neutrophil extracellular traps (NETs) (29, 30). Some studies suggest circulating neutrophils may be an important contributor to AAA formation in the early phase. Eliason et al. found AAA of wild-type animals (WTs) grew faster than mice with neutropenia 4 days after elastase perfusion to induce AAA, although there was not a significant difference in the 7^th^ day (31). A cohort study showed that there were strong associations between elevated neutrophil counts and AAA (32). Li et al. that identified FAM3D, a novel chemokine, was strikingly upregulated in human AAA tissues, and *Fam3d* ^−/−^ mice had decreased levels of neutrophil infiltration than WTs. Besides, administration of FAM3D neutralizing antibody markedly suppressed AAA expansion (33).

The effective integrant of neutrophils is composed with granules and secretory vesicles consisting of various enzymes (28). There are three kinds of granules within neutrophils in total. The azurophilic granules contain myeloperoxidase (MPO), an enzyme essential for the oxidative burst, and other components including defensins, lysozyme and some proteases such as neutrophil elastase and proteinase 3 (34). The specific (secondary) granules are peroxidase-negative and storage lactoferrin, hCAP18, NGAL, lysozyme, and NRAMP-1 (35). The last type is called gelatinase (tertiary) granules. Although there are very few antimicrobials in gelatinase granules, they contain a host of MMPs (34).

**Figure 1:**
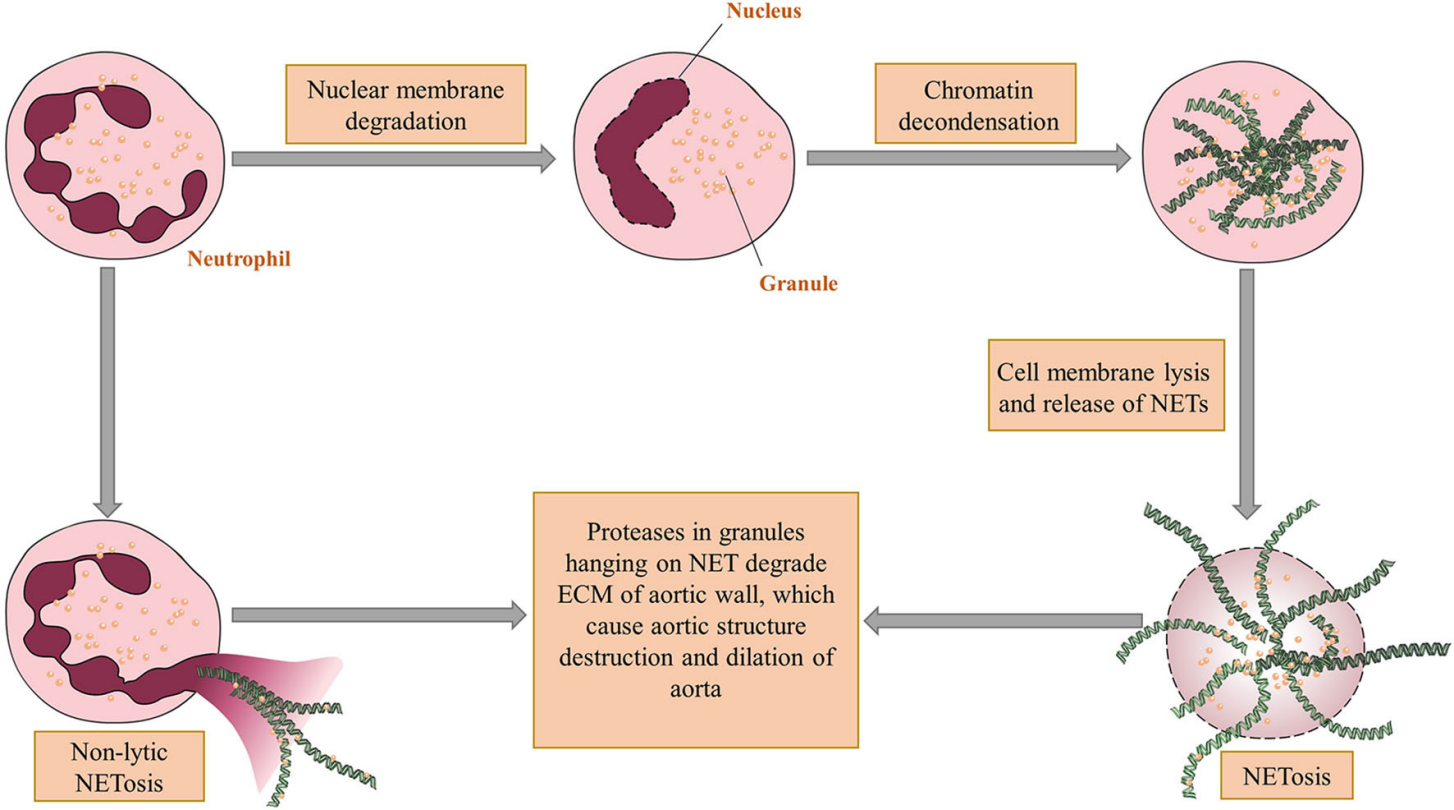
The mechanism of NET formation and acting on aortic walls. There are two ways for neutrophil extracellular traps (NET) come into being. The first one is called NETosis in which nuclei of neutrophils undergo delobulation, chromatin decondensation and nuclear membrane lysis. After that neutrophil granules adhering to released chromatin enter extracellular spaces through ruptured cell membranes. The other way, which is a non-lytic form of NETosis, occurs after partial depolarization of nuclei and render granules hanging on chromatin out of plasma without cell deaths. The proteases within granules can thereby directly degrade the vascular structure and cause aortic dilation. Figures were produced using Servier Medical Art (www.servier.com).

NETs are net-like structures protruding from cell membranes of neutrophils or released from ruptured neutrophils (36). When neutrophils are activated, a process named NETosis (**Figure 1**) initiates. The first way of NETosis starts with nuclear delobulation and decondense of chromatin, followed by cellular depolarization and membrane rupture to release NETs. Another kind of NETosis, which is termed as non-lytic form of NETosis, proceeds with expulsion of chromatin and degranulation (37). NETs may have several impacts on aortic wall. To begin with, the proteases hanging on NETs like MMPs can cause direct damage to aortic walls after chromatin are cleaved by DNases (38). Besides, NETs can increase the transcription of IL-6 and pro-IL-1β in macrophages, induce Th17 cell differentiation and recruit more inflammatory cells (30). Another possible effect of NETs on AAA pathogenesis is promoting vascular occlusion. The net-like structure of NETs can render blood cell gathering within the aorta and finally cause thrombosis (36). NETs also help establish the bridge between neutrophils and other immune cells. Cathelicidin-related antimicrobial peptide exposed by NETs can bind to self-DNA and subsequently recruit plasmacytoid DCs (pDCs) that induce type I interferon synthesis (39).

### Dendritic Cells

Dendritic cells (DCs) are a kind of antigen presenting cells (APC) which are able to process and expose antigen components to T lymphocytes, play a key role in the induction of innate immune responses and are implicated in the immune tolerance to self-antigens (40, 41). Krishna et al. indicated that depletion of CD11c^+^ cells can significantly decrease the maximum diameter of AAAs 28 days after angiotensin II infusion (40), which suggests that DCs may also have important impact on the development of AAA.

DCs generally express CD11c and major histocompatibility (MHC) class II molecules. The four subsets of DCs are conventional DCs (cDCs), Langerhans cells, monocyte-derived DCs and pDCs (42). In that the main resident site of Langerhans cells are the epidermis and mucosa, the effective types of DCs on AAAs are cDCs, monocyte derived DCs and pDCs. All kinds of DCs derive from macrophage and DC precursors (MDP), which give rise to monocytes and the common DC precursors (CDP) (43). CDP could further differentiate into pDCs and pre-cDCs. pDCs are a special DC subset which can promote antiviral responses and are also involved in pathophysiology of autoimmune diseases (44). pDCs are able to produce type I interferons, such as IFN-α and IFN-β, to promote proinflammatory responses through activating effector T cell, cytotoxic T cells, and NK cells (39, 45). These inflammatory cells can further facilitate AAA development. cDC1s and cDC2s are two subsets differentiated from pre-cDCs. cDC1s are well known for their cross-presenting functions, and are involved in immune responses to bacterial and viral infections. cDC2s are specialized for sensing danger signals and producing high levels of IL-6 and IL-8 (46). These two phenotypes of cDCs are both characterized as regulatory mediators of immune responses. cDC1 can activate CD8^+^ T cells, promote T helper type 1 (Th1) activation by MHC class I, and activate natural killer responses with by IL-12 (47, 48). cDC2 can cross-present antigens to induce the proliferation of Th1 cells though MHC class II molecules (49). Their effects enrich the communications in the inflammatory microenvironment of AAA tissues. The process that monocytes differentiate into DCs under the induction of GM-CSF plus IL-4 has been observed *in vitro* culture. Monocyte-derived DCs have the potential to transform into cDCs, and *in vivo* experiments showed they can induce Th1 and Th17 cell polarizations (50). However, the detailed roles of DC subsets in AAA need to be explored.

### Mast Cells

Mast cells are widely distributed in the tunica adventitia and media of aortic wall. The mast cell count is positively correlated with the maximum of AAA diameter (51). The roles of mast cells in AAA have been intensively discussed in Shi et al.’s review, that elevated proteases of mast cells like chymase and tryptase in patients with AAA, and these proteases contribute to leukocyte adhesion and migration, vascular smooth muscle cells (VSMC) apoptosis, foam cell formation, and expression of MMP and cathepsins (52). Cathepsin is a kind of enzyme containing in mast cells. Cathepsin C (*Ctsc*) acts as an upstream activator of tryptases, chymases and other cathepsins by cleaving the N-terminal pro-peptide of the zymogen forms of these proteases (53). Cathepsin G has similar function with chymases, which can generate angiotensin II from angiotensin I. Mice deficient of *Ctsc* were resistant to elastase perfusion-induced AAA compared with WT mice, and suffered from less transmural inflammatory cell infiltration (54). However, controlling mast cells solely are not efficient enough as a medical treatment option for aortic aneurysms. A randomized clinical trial showed that pemirolast, a potential mast cell stabilizer, could not inhibit the development of AAA at several different doses, which may be due to the limited influences of pemirolast on plasma tryptase concentration (55, 56). In addition to directly suppress the activity of mast cells, diminishing their impact like inducing VSMC apoptosis might be an alternative way to treat AAAs. A master regulator of autophagy and lysosome biogenesis named transcription factor EB, for example, was shown to prevent VSMC apoptosis and attenuate AAA development (57).

### Natural Killer Cells

NK cells are lymphocytes which have important effects on innate immune responses to tumors and infections (58). Although the fraction of NK cells is not that high as T cells in AAA tissues, they have an impact on aneurysm development both through causing aortic wall damage and through accelerating atherosclerotic changes (59–61). NKT cells, a special subtype of immune cells that express both T cell receptor and markers characteristic of NK cells, are amplified both *in vivo* and *in vitro* after injected with Ang II. NKT cells exacerbate aneurysm progression by increasing matrix degrading enzymes in VSMC and macrophages, and by secreting cytokine downregulating VSMC viability (62, 63). Forester et al. reveal peripheral level and cytotoxicity of NK cells are increased in AAA patients than control groups, and these NK cells retained amount and cytotoxicity to destruct VSMC even after aneurysm repair (64).

## Adaptive Immune Cells

### CD4^+^ T Cells

The most predominant infiltrated inflammatory cells in AAA specimens are T lymphocytes (65), and the majority are CD4^+^ T cells (mainly helper T cells). The distinct phenotypes and functions of CD4^+^ T cells are summarized in **Table 1**. Depending on surface markers and functions, CD4^+^ T cells can be differentiated into diverse subsets in response to various microenvironment stimuli, including Th1 cells, Th2 cells, Th17 cells, regulatory T cells and follicular helper T (Tfh) cells (66). Specifically, these CD4^+^ T cells express various immune molecules, including αβ T cell receptors, T cell activation markers, memory cell phenotypes (CD45RO^+^CD45R A^–^CD62L^–^), and distinct patterns of cell surface molecules (including CD54, CD31, CD11a, CD29, CD44, CD95, and CD27) (67).

**Table 1:** Differentiation, function, and role of various phenotypes of CD4+ T cells in AAA.

	Th1	Th2	Th17	Treg	Tfh
Activators	IFN-γ, IL-12	IL-2, IL-4	IL-1, IL-6, TGF-β	TGF-β, IL-2	IL-21, Bcl-6
Affiliated cell	Macrophage, CD8^+^ T cell	B cell, eosinophil, mast cell	Neutrophil		B cell
Products	IFN-γ, IL-2 and TNF-β	IL-4, IL-5, IL-6 and IL-10, FasL	IL-17, IL-21, GM-CSF	TGF-β, IL-10, IL-35	CXCR5, IL-21
Role in AAA	Activate macrophage, inhibit collagen synthesis	↓Macrophage cytotoxicity and MMP secretion, ↑VSMC apoptosis	↑Macrophage and neutrophil recruitment	↓T cell proliferation and IFN-γ production, ↓Inflammatory cell chemotaxis, arterial wall remodeling, and angiogenesis	May upregulate autoantibody secretion through assisting B cell proliferation

#### Th1 and Th2 Cells

The most significant effect of CD4^+^ T cells on AAAs rely on cytokine secretions, such as Th1 cytokines (IFN-γ, IL-2 and TNF-β) and Th2 cytokines (IL-4, IL-5, IL-6 and IL-10) (13, 67). Some of these cytokines are associated with macrophage activation, regulation of VSMC apoptosis and direct destruction of aortic walls (68). Deletion of *Il12b* can inhibit macrophage expansion, decrease production of cytokines like IL-6 and TNF-α in the early stage of AAA, and suppress aneurysm development (69). Another research determined a strikingly higher level of circulating IL-4 in patients with AAA than healthy individuals (70). Wanfen et al. showed that aneurysm dilation and MMP secretion were prevented in *Ifng* deficient mice (71).

Th1 cells, Th2 cells also have effects on aortic wall degradation. There are profound interactions between various types of helper T cells and vascular smooth muscle cells (VSMCs) through autoimmunity. Fas ligand (FasL) expressed by Th2 cells are indicated to promote VSMC death (72). Besides, TNF and IFN-γ released by Th1 cells can further inhibit collagen synthesis (73, 74). A study aiming to investigate the interactions among immune cells in AAAs reveals that CD4^+^ T cells could promote VSMC proliferation through direct cell-to-cell contact (60). VSMC, the main cellular constituent of the aortic wall (75), subsequently induce NK cells aggregation and finally result in VSMC apoptosis. Extracellular matrix (ECM) enables artery wall to obtain the blood containing function, and the main component of ECM, especially collagen and elastin, are synthesized and processed by VSMC. Collagen defects can lead to aneurysm rupture, while elastin depletions are associated with continuous dilation (11). All these results demonstrate the essential position of Th1 and Th2 in aneurysmal diseases.

#### Th17 Cells

Th17 cells, the main origin of IL-17, are elevated in AAA tissues (76). IL-17 secreted by Th17 cells mediates a quantity of immune responses like neutrophil recruitments and plays a central part in vascular superoxide production (77). This can sharpen oxidative stress in aortic walls. Oxidative stress is one of the major pathogenic factors of AAA, and a study proved riboflavin (vitamin B2), a kind of antioxidant, could prevent aneurysm formation in rat models (78), which suggests inhibiting oxidative stress by controlling IL-17 synthesis and activity of Th17 cells may be a potential therapeutic target for AAA patients.

Owing to their various cytokines in addition to IL-17, such as IL-17F, IL-21 and granulocyte-macrophage colony-stimulating factor (GM-CSF), Th17 cells have been implicated in several autoimmune diseases, including inflammatory bowel disease, multiple sclerosis and rheumatoid arthritis (79). Therefore, it is rational to anticipate that Th17 cells is also probably of great relevance to AAA. Ashish et al. showed that there is a evidently higher expression of IL-17 in AAAs. Besides, *Il17a* ^-/-^ mice are relatively resistant to AAA, and plasma concentration of inflammatory cytokines are also decreased, which proved the proinflammatory and atherosclerotic properties of IL-17 (76). Wei et al. introduced digoxin to antagonize retinoic acid-related orphan receptor gamma thymus, a master transcription of Th17 cell differentiation, and found out that this can attenuate aneurysm expansion in two different kinds of models with AAA (80). These findings indicate the role of Th17 cells in AAA development.

#### Tfh Cells

Tfh cells express CXCR5, a chemokine receptor that helps guide cells into B cell follicles (81). Tfh cells could provide assistant to B cells activation through autocrine or interactions with B cells, and are essential for formation and maintenance of germinal centers (82). Tfh cells have a role in atherosclerosis. Gaddis et al. found that deletion of *Bcl6*, a transcription factor of Tfh cells, prevented plaque formation in *Ldlr* ^-/-^ murine models (83). This finding suggests decreasing Tfh cells activity may slow down the exacerbation of aneurysms. However, the roles of Tfh cells in AAA need to be established.

#### Regulatory T Cells

Regulatory T (Treg) cells are a specific kind of CD4^+^ T cells which express forkhead box protein 3 (FOXP3) and regulate the effects of other T cell subsets (84). Treg cells have an impact on suppressing local inflammation, and compromised Treg functions may promote AAA growth (85). The suppressive effect is determined by acetylation levels of FOXP3, which is lower in human aneurysm tissue. SIRT1 can specifically regulate the acetylation of FOXP3 (86). Studies have shown that EX-527, an inhibitor of SIRT1, can recover the acetylation levels of FOXP3, increase the number of active Treg cells and bring back their suppressive functions on AAA (86). Zhou et al. found that Treg cells could release IL-10 and thereby suppress inflammatory cell chemotaxis, arterial wall remodeling, and angiogenesis (87). Another study showed that the proportion of Treg cells in peripheral mononuclear cells were markedly decreased in AAA patients than controls (88). The average aortic diameters of *Foxp3* ^-/-^ mice were larger than WTs after CaCl_2_ induction, while infusion of normal Treg cells to *Foxp3* ^-/-^ mice can render their similar aortic size with WTs after CaCl_2_ induction (88). Administration of IL-2 to expanse FOXP3^+^ Treg cells also reduced the incidence and mortality of AAA in *Apoe* ^-/-^ mice with angiotensin II infusion (89). Besides, Treg cells are an essential source of TGF-β, which is a matrix-protecting and anti-inflammatory cytokine in human. Wang et al. concluded that systemic neutralization of TGF-β would increase the activity of MMP-12 and subsequently contributed to aneurysm progression and rupture (90). This growing body of evidence suggests an important role of Treg cells in enhancing inflammation and inducing AAA enlargement.

### CD8^+^ T Cells

CD8^+^ T cells represent a considerable part of adaptive immunity. According to the immune state, CD8^+^ T cells can be generally divided into effector cells and memory cells, which can provide both immediate clearance and long-term protective effect on killing tumor cells and virally infected cells (91). CD8^+^ T cells are found to be elevated in AAA wall and perivascular tissues (92). Zhou et al. indicated that IFN-γ released by CD8^+^ T cells could promote cellular apoptosis *in vivo* and MMP-producing macrophage recruitment (93). CD8^+^ T cells exert versatile impacts on atherosclerosis. Chemokines like MCP-1 and CCL-2, which can induce monocytes infiltration in atherosclerotic lesions, were observed to be deceased in mice depleted of CD8^+^ T cell (94). However, CD8^+^ T cells can promote apoptosis of antigen presenting cells and suppress functions of CD4^+^ T cells, which can resist progression of atherosclerosis (95). This discrepancy may result from production of inflammatory cytokines and lysis of endothelial cells by CD8^+^ T cells. The pro-atherogenic and protective effects of CD8^+^ T cells may also regulate the enlargement of AAA, but need to be further explored.

### γδ T Cells

In contrast to αβ T cells, γδ T cells are independent of MHC class II or β2 microglobulin for development and activation (96), suggesting that they are eligible to generate rapid immune responses in blood. γδ T cells can produce various cytokines including TNF‐α, IL−17, IL‐22, and IFN‐γ (97). Besides, γδ T cells also secrete chemokines, which influence recruitment of other immune cells at the site of inflammation and modulate the function of other innate and adaptive immune cells (97). These features establish distinct role of γδ T cells in sterile and non-sterile inflammation. γδ T cells were found to be present in samples of AAA patients (98), so the special immune properties of γδ T cells may play of role in early stage of aneurysm formation.

### B Cells

B cells serve as essential functional parts in humoral immunity of the adaptive immune system through secreting antibodies. B cell can be divided into three subpopulations, including B1, B2 and regulatory B cells (99). Schaheen et al. discovered that depletion of B1 and B2 cells with anti-CD20 antibody significantly limit AAA growth in animals treated with elastase perfusion or angiotensin II-infusion (45). However, B2 cell refusion was exhibited to ameliorate AAA exacerbation in B cell-deficiency murine models (100). This anomalous phenomenon might be due to upregulation of Treg cells and TGF-β despite of the atherogenic effects of B2 cells (101), and also serves as another proof that AAA is an inflammation-driven disease rather than simple atherosclerotic lesions. The complex impact of B cells on AAA development may need more studies to verify, such as purely B1 cell deficiency murine models.

**Figure 2:**
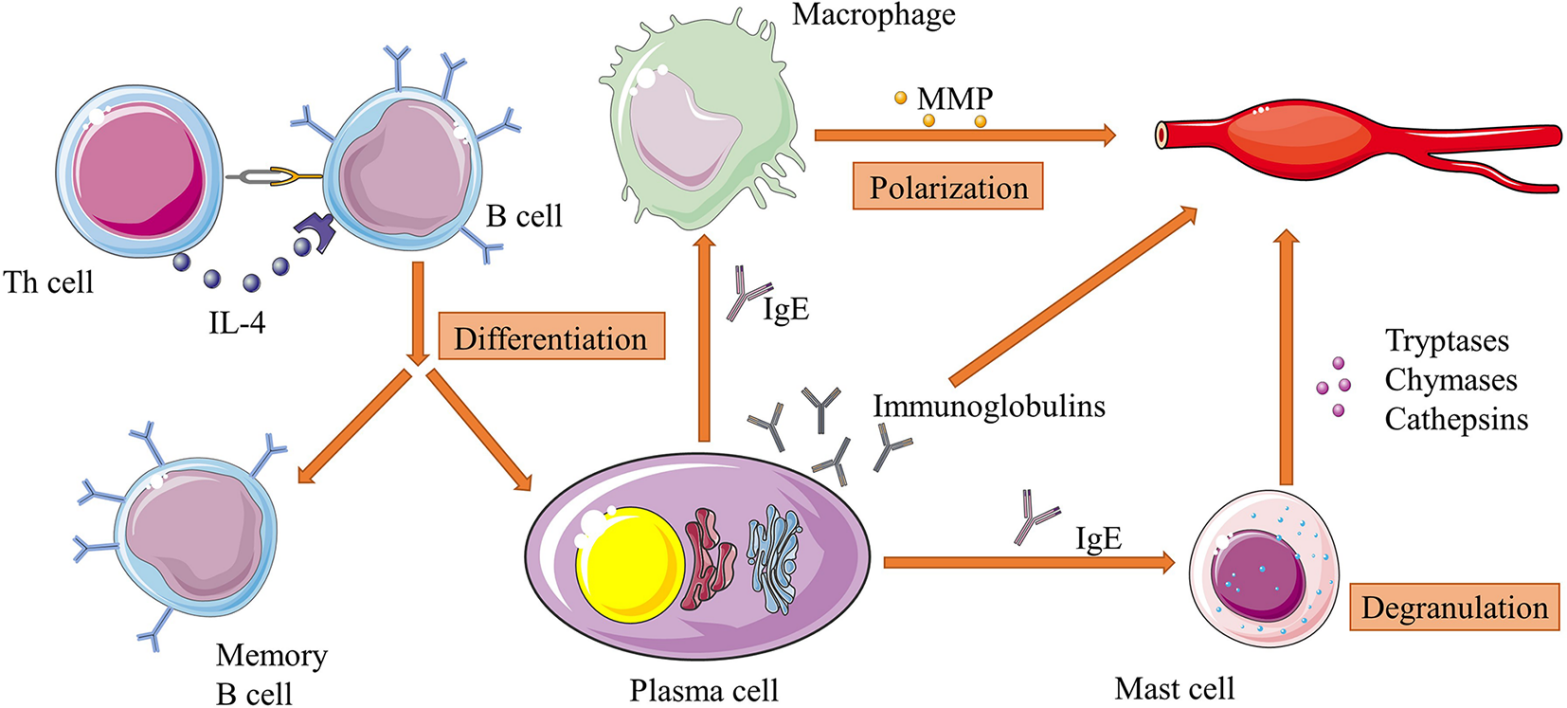
Interactions of between B cells and other immune cells in AAA. B cells can differentiate into plasma cells and memory B cells under the stimulation of IL-4 from Th cells. Plasma cells continuously secrete immunoglobulins, which directly attack aortic walls. Specifically, IgE can activate macrophage polarization and mast cell degranulation and subsequently increase their productions of proteases such as MMPs and cathepsins. These factors work together in the pathogenesis of extracellular matrix degradation of aorta, and is an example of immune cell interactions in the whole process of AAA development. Figures were produced using Servier Medical Art (www.servier.com).

In addition to producing cytokines like TGF-β, the main function of B cells is to secrete immunoglobulins. After contacting with antigens, the activation-induced cytidine deaminase (AID)-driven somatic hypermutation (SHM) of the variable regions of immunoglobulin genes generate a number of mutated B cells that can differentiate into immunoglobulin-secreting plasma cells and memory B cells, which provide both immediate and persistent effects on the same antigens (102). Some of these B cells are overactive and produce autoantibodies after stimulated by autologous components of human tissues, and result in a variety autoimmune diseases including AAA (103, 104). Immunoglobulins were found widely deposited in mouse AAA tissues, and these autoantibodies can not only induce secretions of IL-6 and MMP-9 from T cells and macrophages, but directly cause local destruction of aortic walls (105). For example, B cell-derived anti-beta 2 glycoprotein I antibody was shown to exacerbate HHcy-aggravated vascular inflammation and AAA expansion (106). In addition, a study isolated antiphospholipid (aPL) antibody (a kind of autoantibody able to cause blood clots) from human AAA tissue, and found that more aPL-positive patients underwent AAA progression that aPL-negative patients (107). Another study purified antibodies against Chlamydia pneumoniae outer membrane proteins (OMPs) from serum of AAA patients, and used these antibodies to analyze the aortic walls of AAA patients with western blot and found positive reactions in all of the tested samples, which could be an evidence of the association between the *Chlamydia pneumoniae* OMP antigens and AAA (108). Besides, some of the immunoglobulin subtypes can interact with other immune cells. For instance, IgE can affect macrophage polarization and induce mast cell activated to synthesize various elastases (109, 110). These dramatically increasing evidences indicate that B cell may be an ideal target to treat AAA patients, and subsequent experiments confirmed this hypothesis. Zhang et al. reported that vinpocetine could alleviate AAA development by suppressing TNF-α-induced B cell activation and proinflammatory mediator expression in primary cultured macrophages both *in vitro*, and *in vivo* (111). The interactions of between B cells and other immune cells are illustrated in **Figure 2**.

## Other Inflammatory-Involved Mechanisms

### Matrix Metalloproteinases

MMPs have been implicated in the pathologic origin of AAAs. MMPs have significant destructive effects on elastin fiber integrity, and thereby cause elastin to lose its mechanical properties (112). Several types of MMPs can be secreted by AAA tissue, such as MMP-2, MMP-3, MMP-8, MMP-9, MMP-12 and MMP-13 (113, 114). MMP-9 is the most abundant elastolytic proteinase found in AAA tissue and is predominantly expressed by macrophages infiltrated in AAA (115). Several studies showed that *Mmp9* and *Mmp2* knockout mice are protected from CaCl_2_ challenging, indicating the important role of MMPs in AAA developments (116). Besides, targeted delivery of MMP inhibitors with nanoparticles was shown to inhibit aneurysmal progression (113). Robert et al. found that the relative resistant to AAA formation in *Mmp9* deficient mice was related to the preservative structure of elastic lamellae despite the presence of infiltrating mononuclear phagocytes and neutrophils (115). It has also been found that MMP-9 can hardly cause local tissue injury without the presence of MMP-2, because MMP-2 can initiate cleavage of the triple-helix-structured collagen into one-quarter and three-quarter lengths, which complement the effects of MMP-9 (116). Netrin-1, a neuronal guidance signal that can specifically regulate the activity of MMP-3, was found to be elevated in murine and human AAA tissues, and targeted depletion of *Ntn1* in macrophages evidently decreased the risk of developing murine AAA (117).

All of above mechanisms give MMP the potential to be a target of screening and therapy for AAA patients. As a specific history hallmark of aneurysm formation, fragmentation of ECM by MMPs has been frequently studied to investigate particular biomarkers in AAA patients (118). A meta-analysis including eight case-control studies revealed strikingly increase of circulating MMP-9 levels in AAA patients (119). Hovsepian et al. found that the elevated MMP-9 had a sensitivity of 48% and a specificity of 95% to establish AAA diagnosis (120). Several other types, such as MMP-1, -2, -3, -7,-12 and -13 have been shown to have an increased level accompanied with reduction of their inhibitors by some researchers (121–123). Doxycycline is a kind of tetracycline antibiotic which is capable to suppress a cast of MMPs, and has been shown to be effective in reducing elastin degradation and aneurysm development in murine AAA models (1). Small randomized clinical trials showed doxycycline suppressed the expansion of AAA (124). A meta-analysis, however, concluded that patients with doxycycline prescription had no significant growth rate reduction of aneurysm diameter than control groups (125).

### Inflammasomes

**Figure 3:**
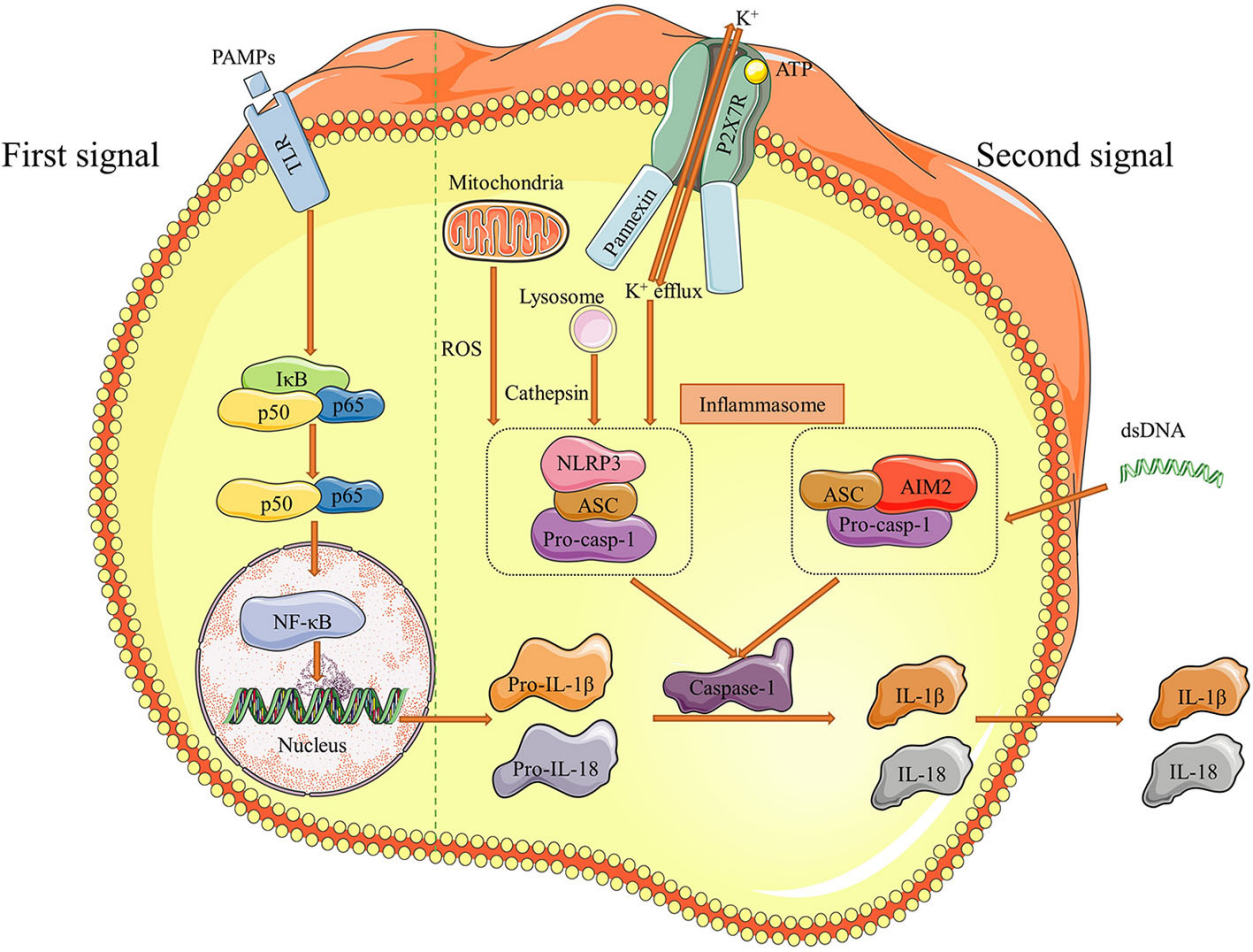
Pathways of NLRP3 and AIM2 inflammasome activation. There are two distinct signals needed for inflammasome to be effective. Initially, pathogen-associated molecular patterns (PAMPs) as the first signal binds to Toll like receptors (TLRs) and stimulate NF-κB, which increases downstream pro-IL-1β and pro-IL-18 production. Then, efflux of K^+^ and dsDNA are the second signals correspondingly to induce NLRP3 and AIM2 inflammasome formation. The pathway of NLRP3 inflammasome activation usually proceed under the assistant of cathepsin released by lysosome and ROS mtDNA from mitochondria. The final result of inflammasome activation is cleaving pro-casp-1 into caspase-1, which transforms pro-IL-1β and pro-IL-18 to IL-1β and IL-18. These two effective cytokines are secreted out and participate the inflammatory responses in aortic walls. Figures were produced using Servier Medical Art (www.servier.com).

Inflammasomes are large multimolecular complexes that are able to induce inflammation reactions and control the activation of caspase-1, which regulates the proteolytic maturation of IL-1β and IL-18 (126, 127). These intracellular molecular protein scaffolds work through inducing pyroptosis (an inflammatory form of cell death) and necroptosis (a lytic form of inflammatory cell death) by cleaving the N-terminal of pro-IL-1β and pro-IL-18 with caspase-1 (128). Five kinds of receptor proteins have been identified so far to assemble inflammasomes, including nucleotide-binding oligomerization domain (NOD), leucine-rich repeat (LRR)-containing protein (NLR) family members NLRP1, NLRP3 and NLRC4, as well as the proteins absent in melanoma 2 (AIM2) and pyrin (126). It has been shown that inflammasomes are involved in a cast of inflammatory disorders (126). Recent works suggest that NLRP3 and AIM2 inflammasomes are implicated in the pathogenesis of AAA, and we summarized the process of these inflammasome activations in **Figure 3**.

A pilot study demonstrated an upregulation of the inflammasome core components ASC (apoptosis associated speck-like protein containing a caspase activation and recruitment domain), caspase-1 and IL-1β in AAA tissue compared to normal aortas and claimed AAA-associated lymphoid cells could carry on inflammasome signaling (129). Some subsets of inflammasomes like AIM2 were significantly increased in circulating granulocytes, monocytes, B lymphocytes of AAA patients, and IL-1β released by peripheral blood mononuclear cells of AAA patients was significantly higher than controls (130). Another study found expression of NLRP3 and AIM2 were notably lower in control samples than AAA. However, with the AAA lesion progression, inflammasome expressions decreased (131), which suggests the inflammasome-induced signaling plays a more important role in early AAA pathogenesis. Markus et al. found that necrotic cell debris from autologous cells promotes AIM2 and NLRP3 inflammasomes in VSMC of late stage AAA tissues, and thereby activates downstream inflammatory attacks (132). Ren, et al. found that NLRP3 inflammasomes directly activate MMP-9 by cleaving its N-terminal inhibitory domain, so blocking the inflammasome pathway with MCC950, a potent selective small-molecule NLRP3-inflammasome inhibitor, could prevent aortic aneurysm formation (133). Similarly, silencing of NLRP3 in macrophages remarkably ameliorated AAA formation (134). In the meanwhile, NLPR3, caspase 1, and IL-1β levels were elevated in hyperhomocysteinemia (HHcy) models compared with WTs, and administration of folic acid to reverse the HHcy-accelerated AAA could alleviate activation of inflammasomes in the tunica adventitia (134). These studies demonstrate inflammasomes may be a promising target for medical intervention of AAA.

## Perspectives

AAA still remains to be a life-threatening disease. In the current review, we summarized the updated pathogenic roles of inflammatory cells in AAA development. The roles of T cells and macrophages in AAA have been predominantly studied, including inflammatory cytokines, MMPs, inflammasomes, etc. However, how the other types of inflammatory cells influence AAA are still not fully verified. Despite of the advances of endovascular aneurysm repair and open surgery for large or ruptured AAA, there is still lacking efficient medical therapy choices for asymptomatic patients. This review lists a considerable number of pathways of inflammatory cell effects, and provides evidences from studies that suppressing corresponding pathways may influence the development of AAA in murine models or patient samples *in vitro*. These evidences not only prove the irreplaceable roles of inflammatory cells in AAA, but provide new methods to develop ideal drugs for researchers and physicians. Specific targets, such as inflammatory cytokines and MMPs, have been investigated for biomarker screening and possible medical therapies for asymptomatic AAA. These novel applications may serve as advanced strategies for early identification and therapeutic intervention for AAA.

It should be noted that most studies on detailed cellular mechanisms were conducted in animal models or *in vitro* experiments, which could not entirely mimic the pathogenesis of AAA in humans. Studies bridging pre-clinical mechanisms and clinical data are needed. Furthermore, most of the animal studies were only focused on the initiation of diseases, while how to prevent AAA rupture in real-world patients are more challenging. Further studies on different stages of AAA will be helpful.

## Author Contributions

YZ, YL, JW, JQW, JY, and ZX drafted, edited, and approved the manuscript and figures. All authors contributed to the article and approved the submitted version.

## Funding

This work was supported by funding from the National Natural Science Foundation of China (81970396 and 81900416), the Zhejiang Provincial Natural Science Foundation for Distinguished Young Scholars (LR20H020002), and the Zhejiang Provincial Natural Science Foundation of China (LQ19H020006 and LY19H040012).

## Conflict of Interest

The authors declare that the research was conducted in the absence of any commercial or financial relationships that could be construed as a potential conflict of interest.

## References

[1] Klink A Hyafil F Rudd J Faries P Fuster V Mallat Z Diagnostic and therapeutic strategies for small abdominal aortic aneurysms. Nat Rev Cardiol (2011) 8(6):338–47. 10.1038/nrcardio.2011.1 21304473

[2] Umebayashi R Uchida HA Wada J Abdominal aortic aneurysm in aged population. Aging (Albany NY) (2018) 10(12):3650–1. 10.18632/aging.101702 PMC6326692 30523221

[3] Lindquist Liljeqvist M Hultgren R Bergman O Villard C Kronqvist M Eriksson P Tunica-Specific Transcriptome of Abdominal Aortic Aneurysm and the Effect of Intraluminal Thrombus, Smoking, and Diameter Growth Rate. Arterioscler Thromb Vasc Biol (2020) 40(11):2700–13. 10.1161/ATVBAHA.120.314264 32907367

[4] Arnaoutakis DJ Upchurch GR Jr. Abdominal Aortic Aneurysm Screening Is Safe yet Lacks Effectiveness. Circulation (2019) 139(11):1381–3. 10.1161/CIRCULATIONAHA.118.038809 30855994

[5] Eckstein HH Bockler D Flessenkamper I Schmitz-Rixen T Debus S Lang W Ultrasonographic screening for the detection of abdominal aortic aneurysms. Dtsch Arztebl Int (2009) 106(41):657–63. 10.3238/arztebl.2009.0657 PMC2780009 19946430

[6] Zankl AR Schumacher H Krumsdorf U Katus HA Jahn L Tiefenbacher CP Pathology, natural history and treatment of abdominal aortic aneurysms. Clin Res Cardiol (2007) 96(3):140–51. 10.1007/s00392-007-0472-5 17180573

[7] Brady AR Thompson SG Fowkes FG Greenhalgh RM Powell JT Participants UKSAT. Abdominal aortic aneurysm expansion: risk factors and time intervals for surveillance. Circulation (2004) 110(1):16–21. 10.1161/01.CIR.0000133279.07468.9F 15210603

[8] Tilson MD Decline of the atherogenic theory of the etiology of the abdominal aortic aneurysm and rise of the autoimmune hypothesis. J Vasc Surg (2016) 64(5):1523–5. 10.1016/j.jvs.2016.06.119 27633167

[9] Sakalihasan N Limet R Defawe OD Abdominal aortic aneurysm. Lancet (2005) 365(9470):1577–89. 10.1016/S0140-6736(05)66459-8 15866312

[10] Piacentini L Werba JP Bono E Saccu C Tremoli E Spirito R Genome-Wide Expression Profiling Unveils Autoimmune Response Signatures in the Perivascular Adipose Tissue of Abdominal Aortic Aneurysm. Arterioscler Thromb Vasc Biol (2019) 39(2):237–49. 10.1161/ATVBAHA.118.311803 30567485

[11] Sakalihasan N Michel JB Katsargyris A Kuivaniemi H Defraigne JO Nchimi A Abdominal aortic aneurysms. Nat Rev Dis Primers (2018) 4(1):34. 10.1038/s41572-018-0030-7 30337540

[12] Cameron SJ Russell HM Owens AP 3rd Antithrombotic therapy in abdominal aortic aneurysm: beneficial or detrimental? Blood (2018) 132(25):2619–28. 10.1182/blood-2017-08-743237 PMC6302498 30228233

[13] Chang TW Gracon AS Murphy MP Wilkes DS Exploring autoimmunity in the pathogenesis of abdominal aortic aneurysms. Am J Physiol Heart Circ Physiol (2015) 309(5):H719–27. 10.1152/ajpheart.00273.2015 26116712

[14] Nosalski R Guzik TJ Perivascular adipose tissue inflammation in vascular disease. Br J Pharmacol (2017) 174(20):3496–513. 10.1111/bph.13705 PMC5610164 28063251

[15] Guerriero JL Macrophages: Their Untold Story in T Cell Activation and Function. Int Rev Cell Mol Biol (2019) 342:73–93. 10.1016/bs.ircmb.2018.07.001 30635094

[16] Xie S Ma L Guan H Guan S Wen L Han C Daphnetin suppresses experimental abdominal aortic aneurysms in mice via inhibition of aortic mural inflammation. Exp Ther Med (2020) 20(6):221. 10.3892/etm.2020.9351 33193836 PMC7646695

[17] Yan H Cui B Zhang X Fu X Yan J Wang X Antagonism of toll-like receptor 2 attenuates the formation and progression of abdominal aortic aneurysm. Acta Pharm Sin B (2015) 5(3):176–87. 10.1016/j.apsb.2015.03.007 PMC4629243 26579444

[18] Ginhoux F Guilliams M Tissue-Resident Macrophage Ontogeny and Homeostasis. Immunity (2016) 44(3):439–49. 10.1016/j.immuni.2016.02.024 26982352

[19] Zhao G Lu H Chang Z Zhao Y Zhu T Chang L Single cell RNA sequencing reveals the cellular heterogeneity of aneurysmal infrarenal abdominal aorta. Cardiovasc Res (2020) cvaa214. 10.1093/cvr/cvaa214 PMC8064434 32678909

[20] Raffort J Lareyre F Clement M Hassen-Khodja R Chinetti G Mallat Z Monocytes and macrophages in abdominal aortic aneurysm. Nat Rev Cardiol (2017) 14(8):457–71. 10.1038/nrcardio.2017.52 28406184

[21] Kratofil RM Kubes P Deniset JF Monocyte Conversion During Inflammation and Injury. Arterioscler Thromb Vasc Biol (2017) 37(1):35–42. 10.1161/ATVBAHA.116.308198 27765768

[22] Lawrence T Natoli G Transcriptional regulation of macrophage polarization: enabling diversity with identity. Nat Rev Immunol (2011) 11(11):750–61. 10.1038/nri3088 22025054

[23] Funes SC Rios M Escobar-Vera J Kalergis AM Implications of macrophage polarization in autoimmunity. Immunology (2018) 154(2):186–95. 10.1111/imm.12910 PMC5980179 29455468

[24] Ivashkiv LB IFNgamma: signalling, epigenetics and roles in immunity, metabolism, disease and cancer immunotherapy. Nat Rev Immunol (2018) 18(9):545–58. 10.1038/s41577-018-0029-z PMC6340644 29921905

[25] Koelwyn GJ Corr EM Erbay E Moore KJ Regulation of macrophage immunometabolism in atherosclerosis. Nat Immunol (2018) 19(6):526–37. 10.1038/s41590-018-0113-3 PMC6314674 29777212

[26] Gordon S Martinez FO Alternative activation of macrophages: mechanism and functions. Immunity (2010) 32(5):593–604. 10.1016/j.immuni.2010.05.007 20510870

[27] Cheng J Koenig SN Kuivaniemi HS Garg V Hans CP Pharmacological inhibitor of notch signaling stabilizes the progression of small abdominal aortic aneurysm in a mouse model. J Am Heart Assoc (2014) 3(6):e001064. 10.1161/JAHA.114.001064 25349182 PMC4338693

[28] Kolaczkowska E Kubes P Neutrophil recruitment and function in health and inflammation. Nat Rev Immunol (2013) 13(3):159–75. 10.1038/nri3399 23435331

[29] Liew PX Kubes P The Neutrophil’s Role During Health and Disease. Physiol Rev (2019) 99(2):1223–48. 10.1152/physrev.00012.2018 30758246

[30] Papayannopoulos V Neutrophil extracellular traps in immunity and disease. Nat Rev Immunol (2018) 18(2):134–47. 10.1038/nri.2017.105 28990587

[31] Eliason JL Hannawa KK Ailawadi G Sinha I Ford JW Deogracias MP Neutrophil depletion inhibits experimental abdominal aortic aneurysm formation. Circulation (2005) 112(2):232–40. 10.1161/CIRCULATIONAHA.104.517391 16009808

[32] Shah AD Denaxas S Nicholas O Hingorani AD Hemingway H Neutrophil Counts and Initial Presentation of 12 Cardiovascular Diseases: A CALIBER Cohort Study. J Am Coll Cardiol (2017) 69(9):1160–9. 10.1016/j.jacc.2016.12.022 PMC5332591 28254179

[33] He L Fu Y Deng J Shen Y Wang Y Yu F Deficiency of FAM3D (Family With Sequence Similarity 3, Member D), A Novel Chemokine, Attenuates Neutrophil Recruitment and Ameliorates Abdominal Aortic Aneurysm Development. Arterioscler Thromb Vasc Biol (2018) 38(7):1616–31. 10.1161/ATVBAHA.118.311289 PMC6039426 29853563

[34] Amulic B Cazalet C Hayes GL Metzler KD Zychlinsky A Neutrophil function: from mechanisms to disease. Annu Rev Immunol (2012) 30:459–89. 10.1146/annurev-immunol-020711-074942 22224774

[35] Faurschou M Borregaard N Neutrophil granules and secretory vesicles in inflammation. Microbes Infect (2003) 5(14):1317–27. 10.1016/j.micinf.2003.09.008 14613775

[36] Doring Y Soehnlein O Weber C Neutrophil Extracellular Traps in Atherosclerosis and Atherothrombosis. Circ Res (2017) 120(4):736–43. 10.1161/CIRCRESAHA.116.309692 28209798

[37] Sollberger G Tilley DO Zychlinsky A Neutrophil Extracellular Traps: The Biology of Chromatin Externalization. Dev Cell (2018) 44(5):542–53. 10.1016/j.devcel.2018.01.019 29533770

[38] Lee KH Kronbichler A Park DD Park Y Moon H Kim H Neutrophil extracellular traps (NETs) in autoimmune diseases: A comprehensive review. Autoimmune Rev (2017) 16(11):1160–73. 10.1016/j.autrev.2017.09.012 28899799

[39] Yan H Zhou HF Akk A Hu Y Springer LE Ennis TL Neutrophil Proteases Promote Experimental Abdominal Aortic Aneurysm via Extracellular Trap Release and Plasmacytoid Dendritic Cell Activation. Arterioscler Thromb Vasc Biol (2016) 36(8):1660–9. 10.1161/ATVBAHA.116.307786 PMC4965335 27283739

[40] Krishna SM Moran CS Jose RJ Lazzaroni S Huynh P Golledge J Depletion of CD11c+ dendritic cells in apolipoprotein E-deficient mice limits angiotensin II-induced abdominal aortic aneurysm formation and growth. Clin Sci (Lond) (2019) 133(21):2203–15. 10.1042/CS20190924 31696215

[41] Bobryshev YV Lord RS Vascular-associated lymphoid tissue (VALT) involvement in aortic aneurysm. Atherosclerosis (2001) 154(1):15–21. 10.1016/S0021-9150(00)00441-X 11137078

[42] Pearce EJ Everts B Dendritic cell metabolism. Nat Rev Immunol (2015) 15(1):18–29. 10.1038/nri3771 25534620 PMC4495583

[43] Patente TA Pinho MP Oliveira AA Evangelista GCM Bergami-Santos PC Barbuto JAM Human Dendritic Cells: Their Heterogeneity and Clinical Application Potential in Cancer Immunotherapy. Front Immunol (2018) 9:3176. 10.3389/fimmu.2018.03176 30719026 PMC6348254

[44] Swiecki M Colonna M The multifaceted biology of plasmacytoid dendritic cells. Nat Rev Immunol (2015) 15(8):471–85. 10.1038/nri3865 PMC4808588 26160613

[45] Schaheen B Downs EA Serbulea V Almenara CC Spinosa M Su G B-Cell Depletion Promotes Aortic Infiltration of Immunosuppressive Cells and Is Protective of Experimental Aortic Aneurysm. Arterioscler Thromb Vasc Biol (2016) 36(11):2191–202. 10.1161/ATVBAHA.116.307559 PMC5083246 27634836

[46] Balan S Saxena M Bhardwaj N Dendritic cell subsets and locations. Int Rev Cell Mol Biol (2019) 348:1–68. 10.1016/bs.ircmb.2019.07.004 31810551

[47] Collin M Bigley V Human dendritic cell subsets: an update. Immunology (2018) 154(1):3–20. 10.1111/imm.12888 29313948 PMC5904714

[48] Ferris ST Durai V Wu R Theisen DJ Ward JP Bern MD cDC1 prime and are licensed by CD4(+) T cells to induce anti-tumour immunity. Nature (2020) 584(7822):624–9. 10.1038/s41586-020-2611-3 PMC7469755 32788723

[49] Binnewies M Mujal AM Pollack JL Combes AJ Hardison EA Barry KC Unleashing Type-2 Dendritic Cells to Drive Protective Antitumor CD4(+) T Cell Immunity. Cell (2019) 177(3):556–71.e16. 10.1016/j.cell.2019.02.005 30955881 PMC6954108

[50] León B López-Bravo M Ardavín C Monocyte-derived dendritic cells. Semin Immunol (2005) 17(4):313–8. 10.1016/j.smim.2005.05.013 15955712

[51] Tsuruda T Kato J Hatakeyama K Kojima K Yano M Yano Y Adventitial mast cells contribute to pathogenesis in the progression of abdominal aortic aneurysm. Circ Res (2008) 102(11):1368–77. 10.1161/CIRCRESAHA.108.173682 18451339

[52] Wang Y Shi GP Mast cell chymase and tryptase in abdominal aortic aneurysm formation. Trends Cardiovasc Med (2012) 22(6):150–5. 10.1016/j.tcm.2012.07.012 PMC3489993 22902093

[53] Caughey GH Mast cell proteases as pharmacological targets. Eur J Pharmacol (2016) 778:44–55. 10.1016/j.ejphar.2015.04.045 25958181 PMC4636979

[54] Pagano MB Bartoli MA Ennis TL Mao D Simmons PM Thompson RW Critical role of dipeptidyl peptidase I in neutrophil recruitment during the development of experimental abdominal aortic aneurysms. Proc Natl Acad Sci U S A (2007) 104(8):2855–60. 10.1073/pnas.0606091104 PMC1797622 17301245

[55] Sillesen H Eldrup N Hultgren R Lindeman J Bredahl K Thompson M Randomized clinical trial of mast cell inhibition in patients with a medium-sized abdominal aortic aneurysm. Br J Surg (2015) 102(8):894–901. 10.1002/bjs.9824 25963302

[56] Golledge J Moxon JV Singh TP Bown MJ Mani K Wanhainen A Lack of an effective drug therapy for abdominal aortic aneurysm. J Intern Med (2020) 288(1):6–22. 10.1111/joim.12958 31278799

[57] Lu H Sun J Liang W Chang Z Rom O Zhao Y Cyclodextrin Prevents Abdominal Aortic Aneurysm via Activation of Vascular Smooth Muscle Cell Transcription Factor EB. Circulation (2020) 142(5):483–98. 10.1161/CIRCULATIONAHA.119.044803 PMC7606768 32354235

[58] O’Brien KL Finlay DK Immunometabolism and natural killer cell responses. Nat Rev Immunol (2019) 19(5):282–90. 10.1038/s41577-019-0139-2 30808985

[59] Patel A Jagadesham VP Porter KE Scott DJ Carding SR Characterisation of fractalkine/CX3CL1 and fractalkine receptor (CX3CR1) expression in abdominal aortic aneurysm disease. Eur J Vasc Endovasc Surg (2008) 36(1):20–7. 10.1016/j.ejvs.2008.01.014 18296082

[60] Chan WL Pejnovic N Hamilton H Liew TV Popadic D Poggi A Atherosclerotic abdominal aortic aneurysm and the interaction between autologous human plaque-derived vascular smooth muscle cells, type 1 NKT, and helper T cells. Circ Res (2005) 96(6):675–83. 10.1161/01.RES.0000160543.84254.f1 15731463

[61] Biros E Moran CS Rush CM Gäbel G Schreurs C Lindeman JH Differential gene expression in the proximal neck of human abdominal aortic aneurysm. Atherosclerosis (2014) 233(1):211–8. 10.1016/j.atherosclerosis.2013.12.017 24529146

[62] van Puijvelde GHM Foks AC van Bochove RE Bot I Habets KLL de Jager SC CD1d deficiency inhibits the development of abdominal aortic aneurysms in LDL receptor deficient mice. PLoS One (2018) 13(1):e0190962. 10.1371/journal.pone.0190962 29346401 PMC5773169

[63] Hinterseher I Schworer CM Lillvis JH Stahl E Erdman R Gatalica Z Immunohistochemical analysis of the natural killer cell cytotoxicity pathway in human abdominal aortic aneurysms. Int J Mol Sci (2015) 16(5):11196–212. 10.3390/ijms160511196 PMC4463696 25993291

[64] Forester ND Cruickshank SM Scott DJ Carding SR Increased natural killer cell activity in patients with an abdominal aortic aneurysm. Br J Surg (2006) 93(1):46–54. 10.1002/bjs.5215 16315339

[65] Koch AE Haines GK Rizzo RJ Radosevich JA Pope RM Robinson PG Human abdominal aortic aneurysms. Immunophenotypic analysis suggesting an immune-mediated response. Am J Pathol (1995) 137(5):1199–213. PMC1877681 1700620

[66] Zhou L Chong MM Littman DR Plasticity of CD4+ T cell lineage differentiation. Immunity (2009) 30(5):646–55. 10.1016/j.immuni.2009.05.001 19464987

[67] Curci JA Thompson RW Adaptive cellular immunity in aortic aneurysms: cause, consequence, or context? J Clin Invest (2004) 114(2):168–71. 10.1172/JCI22309 PMC449753 15254583

[68] Lindholt JS Shi GP Chronic inflammation, immune response, and infection in abdominal aortic aneurysms. Eur J Vasc Endovasc Surg (2006) 31(5):453–63. 10.1016/j.ejvs.2005.10.030 16414293

[69] Yan H Hu Y Akk A Ye K Bacon J Pham CTN Interleukin-12 and -23 blockade mitigates elastase-induced abdominal aortic aneurysm. Sci Rep (2019) 9(1):10447. 10.1038/s41598-019-46909-y 31320700 PMC6639297

[70] Jablonska A Neumayer C Bolliger M Gollackner B Klinger M Paradowska E Analysis of host Toll-like receptor 3 and RIG-I-like receptor gene expression in patients with abdominal aortic aneurysm. J Vasc Surg (2018) 68(6S):39S–46S. 10.1016/j.jvs.2017.10.087 29567028

[71] Xiong W Zhao Y Prall A Greiner TC Baxter BT Key roles of CD4+ T cells and IFN-gamma in the development of abdominal aortic aneurysms in a murine model. J Immunol (2004) 172(4):2607–12. 10.4049/jimmunol.172.4.2607 14764734

[72] Schönbeck U Sukhova GK Gerdes N Libby P TH2 Predominant Immune Responses Prevail in Human Abdominal Aortic Aneurysm. Am J Pathol (2002) 161(2):499–506. 10.1016/S0002-9440(10)64206-X 12163375 PMC1850720

[73] Hellenthal FA Buurman WA Wodzig WK Schurink GW Biomarkers of abdominal aortic aneurysm progression. Part 2: inflammation. Nat Rev Cardiol (2009) 6(8):543–52. 10.1038/nrcardio.2009.102 19546866

[74] Shimizu K Mitchell RN Libby P Inflammation and cellular immune responses in abdominal aortic aneurysms. Arterioscler Thromb Vasc Biol (2006) 26(5):987–94. 10.1161/01.ATV.0000214999.12921.4f 16497993

[75] Quintana RA Taylor WR Cellular Mechanisms of Aortic Aneurysm Formation. Circ Res (2019) 124(4):607–18. 10.1161/CIRCRESAHA.118.313187 PMC6383789 30763207

[76] Sharma AK Lu G Jester A Johnston WF Zhao Y Hajzus VA Experimental abdominal aortic aneurysm formation is mediated by IL-17 and attenuated by mesenchymal stem cell treatment. Circulation (2012) 126(11 Suppl 1):S38–45. 10.1161/CIRCULATIONAHA.111.083451 PMC3448933 22965992

[77] Dale MA Ruhlman MK Baxter BT Inflammatory cell phenotypes in AAAs: their role and potential as targets for therapy. Arterioscler Thromb Vasc Biol (2015) 35(8):1746–55. 10.1161/ATVBAHA.115.305269 PMC4514552 26044582

[78] Yu Z Morimoto K Yu J Bao W Okita Y Okada K Endogenous superoxide dismutase activation by oral administration of riboflavin reduces abdominal aortic aneurysm formation in rats. J Vasc Surg (2016) 64(3):737–45. 10.1016/j.jvs.2015.03.045 26070605

[79] Yang J Sundrud MS Skepner J Yamagata T Targeting Th17 cells in autoimmune diseases. Trends Pharmacol Sci (2014) 35(10):493–500. 10.1016/j.tips.2014.07.006 25131183

[80] Wei Z Wang Y Zhang K Liao Y Ye P Wu J Inhibiting the Th17/IL-17A-related inflammatory responses with digoxin confers protection against experimental abdominal aortic aneurysm. Arterioscler Thromb Vasc Biol (2014) 34(11):2429–38. 10.1161/ATVBAHA.114.304435 25234817

[81] Schmitt N Bentebibel SE Ueno H Phenotype and functions of memory Tfh cells in human blood. Trends Immunol (2014) 35(9):436–42. 10.1016/j.it.2014.06.002 PMC4152409 24998903

[82] Crotty S Follicular helper CD4 T cells (TFH). Annu Rev Immunol (2011) 29:621–63. 10.1146/annurev-immunol-031210-101400 21314428

[83] Gaddis DE Padgett LE Wu R McSkimming C Romines V Taylor AM Apolipoprotein AI prevents regulatory to follicular helper T cell switching during atherosclerosis. Nat Commun (2018) 9(1):1095. 10.1038/s41467-018-03493-5 29545616 PMC5854619

[84] Barbi J Pardoll D Pan F Treg functional stability and its responsiveness to the microenvironment. Immunol Rev (2014) 259(1):115–39. 10.1111/imr.12172 PMC3996455 24712463

[85] Meng X Yang J Dong M Zhang K Tu E Gao Q Regulatory T cells in cardiovascular diseases. Nat Rev Cardiol (2016) 13(3):167–79. 10.1038/nrcardio.2015.169 PMC11849084 26525543

[86] Jiang H Xin S Yan Y Lun Y Yang X Zhang J Abnormal acetylation of FOXP3 regulated by SIRT-1 induces Treg functional deficiency in patients with abdominal aortic aneurysms. Atherosclerosis (2018) 271:182–92. 10.1016/j.atherosclerosis.2018.02.001 29524861

[87] Zhou Y Wu W Lindholt JS Sukhova GK Libby P Yu X Regulatory T cells in human and angiotensin II-induced mouse abdominal aortic aneurysms. Cardiovasc Res (2015) 107(1):98–107. 10.1093/cvr/cvv119 25824145 PMC4560044

[88] Suh MK Batra R Carson JS Xiong W Dale MA Meisinger T Ex vivo expansion of regulatory T cells from abdominal aortic aneurysm patients inhibits aneurysm in humanized murine model. J Vasc Surg (2020) 72(3):1087–96 e1. 10.1016/j.jvs.2019.08.285 31980239 PMC10690961

[89] Yodoi K Yamashita T Sasaki N Kasahara K Emoto T Matsumoto T Foxp3+ regulatory T cells play a protective role in angiotensin II-induced aortic aneurysm formation in mice. Hypertension (2015) 65(4):889–95. 10.1161/HYPERTENSIONAHA.114.04934 25601931

[90] Wang Y Ait-Oufella H Herbin O Bonnin P Ramkhelawon B Taleb S TGF-beta activity protects against inflammatory aortic aneurysm progression and complications in angiotensin II-infused mice. J Clin Invest (2010) 120(2):422–32. 10.1172/JCI38136 PMC2810071 20101093

[91] Henning AN Roychoudhuri R Restifo NP Epigenetic control of CD8(+) T cell differentiation. Nat Rev Immunol (2018) 18(5):340–56. 10.1038/nri.2017.146 PMC6327307 29379213

[92] Sagan A Mikolajczyk TP Mrowiecki W MacRitchie N Daly K Meldrum A T Cells Are Dominant Population in Human Abdominal Aortic Aneurysms and Their Infiltration in the Perivascular Tissue Correlates With Disease Severity. Front Immunol (2019) 10:1979. 10.3389/fimmu.2019.01979 31552015 PMC6736986

[93] Zhou HF Yan H Cannon JL Springer LE Green JM Pham CT CD43-mediated IFN-gamma production by CD8+ T cells promotes abdominal aortic aneurysm in mice. J Immunol (2013) 190(10):5078–85. 10.4049/jimmunol.1203228 PMC3647012 23585675

[94] Cochain C Zernecke A Protective and pathogenic roles of CD8(+) T cells in atherosclerosis. Basic Res Cardiol (2016) 111(6):71. 10.1007/s00395-016-0589-7 27783202

[95] van Duijn J Kuiper J Slutter B The many faces of CD8+ T cells in atherosclerosis. Curr Opin Lipidol (2018) 29(5):411–6. 10.1097/MOL.0000000000000541 30020198

[96] He Y Wu K Hu Y Sheng L Tie R Wang B γδ T cell and other immune cells crosstalk in cellular immunity. J Immunol Res (2014) 2014:960252. 10.1155/2014/960252 24741636 PMC3987930

[97] Paul S Shilpi Lal G Role of gamma-delta (γδ) T cells in autoimmunity. J Leukoc Biol (2015) 97(2):259–71. 10.1189/jlb.3RU0914-443R 25502468

[98] Platsoucas CD Lu S Nwaneshiudu I Solomides C Agelan A Ntaoula N Abdominal aortic aneurysm is a specific antigen-driven T cell disease. Ann N Y Acad Sci (2006) 1085:224–35. 10.1196/annals.1383.019 17182939

[99] Wang Y Liu J Burrows PD Wang JY B Cell Development and Maturation. Adv Exp Med Biol (2020) 1254:1–22. 10.1007/978-981-15-3532-1_1 32323265

[100] Meher AK Johnston WF Lu G Pope NH Bhamidipati CM Harmon DB B2 cells suppress experimental abdominal aortic aneurysms. Am J Pathol (2014) 184(11):3130–41. 10.1016/j.ajpath.2014.07.006 PMC4215033 25194661

[101] Kyaw T Tipping P Bobik A Toh BH Opposing roles of B lymphocyte subsets in atherosclerosis. Autoimmunity (2017) 50(1):52–6. 10.1080/08916934.2017.1280669 28166680

[102] Mesin L Ersching J Victora GD Germinal Center B Cell Dynamics. Immunity (2016) 45(3):471–82. 10.1016/j.immuni.2016.09.001 PMC5123673 27653600

[103] Kasashima S Zen Y IgG4-related inflammatory abdominal aortic aneurysm. Curr Opin Rheumatol (2011) 23(1):18–23. 10.1097/BOR.0b013e32833ee95f 21124083

[104] Shi Y Yang CQ Wang SW Li W Li J Wang SM Characterization of Fc gamma receptor IIb expression within abdominal aortic aneurysm. Biochem Biophys Res Commun (2017) 485(2):295–300. 10.1016/j.bbrc.2017.02.088 28223220

[105] Furusho A Aoki H Ohno-Urabe S Nishihara M Hirakata S Nishida N Involvement of B Cells, Immunoglobulins, and Syk in the Pathogenesis of Abdominal Aortic Aneurysm. J Am Heart Assoc (2018) 7(6). 10.1161/JAHA.117.007750 PMC5907549 29545260

[106] Shao F Miao Y Zhang Y Han L Ma X Deng J B cell-derived anti-beta 2 glycoprotein I antibody contributes to hyperhomocysteinaemia-aggravated abdominal aortic aneurysm. Cardiovasc Res (2020) 116(11):1897–909. 10.1093/cvr/cvz288 31782769

[107] Duftner C Seiler R Dejaco C Chemelli-Steingruber I Schennach H Klotz W Antiphospholipid antibodies predict progression of abdominal aortic aneurysms. PLoS One (2014) 9(6):e99302. 10.1371/journal.pone.0099302 24979700 PMC4076179

[108] Lindholt JS Stovring J Ostergaard L Urbonavicius S Henneberg EW Honore B Serum antibodies against Chlamydia pneumoniae outer membrane protein cross-react with the heavy chain of immunoglobulin in the wall of abdominal aortic aneurysms. Circulation (2004) 109(17):2097–102. 10.1161/01.CIR.0000127772.58427.7E 15117850

[109] Zhang X Li J Luo S Wang M Huang Q Deng Z IgE Contributes to Atherosclerosis and Obesity by Affecting Macrophage Polarization, Macrophage Protein Network, and Foam Cell Formation. Arterioscler Thromb Vasc Biol (2020) 40(3):597–610. 10.1161/ATVBAHA.119.313744 31996021 PMC7047522

[110] Kashiwakura J Otani IM Kawakami T Monomeric IgE and mast cell development, survival and function. Adv Exp Med Biol (2011) 716:29–46. 10.1007/978-1-4419-9533-9_3 21713650

[111] Zhang C Hsu CG Mohan A Shi H Li D Yan C Vinpocetine protects against the development of experimental abdominal aortic aneurysms. Clin Sci (Lond) (2020) 134(22):2959–76. 10.1042/CS20201057 PMC7720778 33111936

[112] Basalyga DM Simionescu DT Xiong W Baxter BT Starcher BC Vyavahare NR Elastin degradation and calcification in an abdominal aorta injury model: role of matrix metalloproteinases. Circulation (2004) 110(22):3480–7. 10.1161/01.CIR.0000148367.08413.E9 PMC1262646 15545515

[113] Nosoudi N Nahar-Gohad P Sinha A Chowdhury A Gerard P Carsten CG Prevention of abdominal aortic aneurysm progression by targeted inhibition of matrix metalloproteinase activity with batimastat-loaded nanoparticles. Circ Res (2015) 117(11):e80–9. 10.1161/CIRCRESAHA.115.307207 PMC4636940 26443597

[114] Abdul-Hussien H Hanemaaijer R Kleemann R Verhaaren BF van Bockel JH Lindeman JH The pathophysiology of abdominal aortic aneurysm growth: corresponding and discordant inflammatory and proteolytic processes in abdominal aortic and popliteal artery aneurysms. J Vasc Surg (2010) 51(6):1479–87. 10.1016/j.jvs.2010.01.057 20488324

[115] Pyo R Lee JK Shipley JM Curci JA Mao D Ziporin SJ Targeted gene disruption of matrix metalloproteinase-9 (gelatinase B) suppresses development of experimental abdominal aortic aneurysms. J Clin Invest (2000) 105(11):1641–9. 10.1172/JCI8931 PMC300851 10841523

[116] Longo GM Xiong W Greiner TC Zhao Y Fiotti N Baxter BT Matrix metalloproteinases 2 and 9 work in concert to produce aortic aneurysms. J Clin Invest (2002) 110(5):625–32. 10.1172/JCI0215334 PMC151106 12208863

[117] Hadi T Boytard L Silvestro M Alebrahim D Jacob S Feinstein J Macrophage-derived netrin-1 promotes abdominal aortic aneurysm formation by activating MMP3 in vascular smooth muscle cells. Nat Commun (2018) 9(1):5022. 10.1038/s41467-018-07495-1 30479344 PMC6258757

[118] Golledge J Tsao PS Dalman RL Norman PE Circulating markers of abdominal aortic aneurysm presence and progression. Circulation (2008) 118(23):2382–92. 10.1161/CIRCULATIONAHA.108.802074 PMC2752737 19047592

[119] Takagi H Manabe H Kawai N Goto SN Umemoto T Circulating matrix metalloproteinase-9 concentrations and abdominal aortic aneurysm presence: a meta-analysis. Interact Cardiovasc Thorac Surg (2009) 9(3):437–40. 10.1510/icvts.2009.208835 19525292

[120] Hovsepian DM Ziporin SJ Sakurai MK Lee JK Curci JA Thompson RW Elevated Plasma Levels of Matrix Metalloproteinase-9 in Patients with Abdominal Aortic Aneurysms: A Circulating Marker of Degenerative Aneurysm Disease. J Vasc Intervent Radiol (2000) 11(10):1345–52. 10.1016/S1051-0443(07)61315-3 11099248

[121] Stather PW Sidloff DA Dattani N Gokani VJ Choke E Sayers RD Meta-analysis and meta-regression analysis of biomarkers for abdominal aortic aneurysm. Br J Surg (2014) 101(11):1358–72. 10.1002/bjs.9593 25131707

[122] Rabkin SW The Role Matrix Metalloproteinases in the Production of Aortic Aneurysm. Prog Mol Biol Transl Sci (2017) 147:239–65. 10.1016/bs.pmbts.2017.02.002 28413030

[123] Ding R McGuinness CL Burnand KG Sullivan E Smith A Matrix metalloproteinases in the aneurysm wall of patients treated with low-dose doxycycline. Vascular (2005) 13(5):290–7. 10.1258/rsmvasc.13.5.290 16288704

[124] Golledge J Powell JT Medical management of abdominal aortic aneurysm. Eur J Vasc Endovasc Surg (2007) 34(3):267–73. 10.1016/j.ejvs.2007.03.006 17540588

[125] Guessous I Periard D Lorenzetti D Cornuz J Ghali WA The efficacy of pharmacotherapy for decreasing the expansion rate of abdominal aortic aneurysms: a systematic review and meta-analysis. PLoS One (2008) 3(3):e1895. 10.1371/journal.pone.0001895 18365027 PMC2267254

[126] Guo H Callaway JB Ting JP Inflammasomes: mechanism of action, role in disease, and therapeutics. Nat Med (2015) 21(7):677–87. 10.1038/nm.3893 PMC4519035 26121197

[127] Rathinam VA Fitzgerald KA Inflammasome Complexes: Emerging Mechanisms and Effector Functions. Cell (2016) 165(4):792–800. 10.1016/j.cell.2016.03.046 27153493 PMC5503689

[128] Rathinam VAK Chan FK Inflammasome, Inflammation, and Tissue Homeostasis. Trends Mol Med (2018) 24(3):304–18. 10.1016/j.molmed.2018.01.004 PMC6456255 29433944

[129] Dihlmann S Erhart P Mehrabi A Nickkholgh A Lasitschka F Bockler D Increased expression and activation of absent in melanoma 2 inflammasome components in lymphocytic infiltrates of abdominal aortic aneurysms. Mol Med (2014) 20:230–7. 10.2119/molmed.2013.00162 PMC4069270 24618883

[130] Wortmann M Xiao X Wabnitz G Samstag Y Hakimi M Bockler D AIM2 levels and DNA-triggered inflammasome response are increased in peripheral leukocytes of patients with abdominal aortic aneurysm. Inflammation Res (2019) 68(4):337–45. 10.1007/s00011-019-01212-4 30758522

[131] Erhart P Cakmak S Grond-Ginsbach C Hakimi M Bockler D Dihlmann S Inflammasome activity in leucocytes decreases with abdominal aortic aneurysm progression. Int J Mol Med (2019) 44(4):1299–308. 10.3892/ijmm.2019.4307 PMC6713432 31432101

[132] Wortmann M Skorubskaya E Peters AS Hakimi M Bockler D Dihlmann S Necrotic cell debris induces a NF-kappaB-driven inflammasome response in vascular smooth muscle cells derived from abdominal aortic aneurysms (AAA-SMC). Biochem Biophys Res Commun (2019) 511(2):343–9. 10.1016/j.bbrc.2019.02.051 30782482

[133] Ren P Wu D Appel R Zhang L Zhang C Luo W Targeting the NLRP3 Inflammasome With Inhibitor MCC950 Prevents Aortic Aneurysms and Dissections in Mice. J Am Heart Assoc (2020) 9(7):e014044. 10.1161/JAHA.119.014044 32223388 PMC7428617

[134] Sun W Pang Y Liu Z Sun L Liu B Xu M Macrophage inflammasome mediates hyperhomocysteinemia-aggravated abdominal aortic aneurysm. J Mol Cell Cardiol (2015) 81:96–106. 10.1016/j.yjmcc.2015.02.005 25680906

